# A New Histology-Based Prognostic Index for Acute Myeloid Leukemia: Preliminary Results for the “AML Urayasu Classification”

**DOI:** 10.3390/jcm14061989

**Published:** 2025-03-15

**Authors:** Toru Mitsumori, Hideaki Nitta, Haruko Takizawa, Hiroko Iizuka-Honma, Chiho Furuya, Maki Fujishiro, Shigeki Tomita, Akane Hashizume, Tomohiro Sawada, Kazunori Miyake, Mitsuo Okubo, Yasunobu Sekiguchi, Miki Ando, Masaaki Noguchi

**Affiliations:** 1 Department of Hematology, Juntendo University Urayasu Hospital, 2-1-1 Tomioka, Urayasu 279-0021, Japan; t.mitsumori.db@juntendo.ac.jp (T.M.); nitta@juntendo.ac.jp (H.N.); takizawa@juntendo.ac.jp (H.T.); hiiduka@juntendo.ac.jp (H.I.-H.); c-furuya@juntendo.ac.jp (C.F.); 2 Division of Hematology, Juntendo University Juntendo Hospital, Tokyo 113-0033, Japan; m-ando@juntendo.ac.jp; 3 Institute for Environmental and Gender-Specific Medicine, Juntendo University Urayasu Hospital, Urayasu 279-0021, Japan; mfujishi@juntendo.ac.jp; 4 Department of Diagnostic Pathology, Juntendo University Urayasu Hospital, Urayasu 279-0021, Japan; sstomita@juntendo-urayasu.jp (S.T.);; 5 Department of Clinical Laboratory, Juntendo University Urayasu Hospital, Urayasu 279-0021, Japan; sawada@juntendo-urayasu.jp; 6 Department of Clinical Laboratory, Faculty of Medical Sciences, Juntendo University, Tokyo 113-8421, Japan; cpm@juntendo.ac.jp; 7 Laboratory of Blood Transfusion, Juntendo University Urayasu Hospital, Urayasu 279-0021, Japan; mi-okubo@juntendo.ac.jp; 8 Hematology Clinic, Saitama Cancer Center, Saitama 362-0806, Japan; yasu_sek@saitama-pho.jp

**Keywords:** AML, Urayasu classification, prognostic index, P53, MRP1, AKR1B10, AKR1B1

## Abstract

**Background:** This study was aimed at elucidating the mechanisms underlying the development of treatment resistance in patients with acute myeloid leukemia (AML) other than M3 myeloid leukemia in order to devise ways to overcome treatment resistance and improve the treatment outcomes in these patients. **Methods**: For this study, we randomly selected 35 patients with AML who had received combined cytarabine plus idarubicin treatment for new-onset AML at our hospital. We performed immunohistochemical analysis of biopsy specimens obtained from the patients to investigate the expressions of 23 treatment-resistance-related proteins, and retrospectively analyzed the correlations between the expression profiles of the resistance proteins and the patient survival. **Results**: The following four proteins were identified as being particularly significant in relation to treatment resistance and patient prognosis: (1) p53; (2) multidrug resistance-associated protein 1 (MRP1; idarubicin extracellular efflux pump); (3) aldo-keto reductase family 1 member B10 (AKR1B10; idarubicin-inactivating enzyme); and (4) AKR1B1 (competitive inhibitor of AKR1B10). Based on our findings, we propose the following Urayasu classification for AML, which we believe would be very useful for accurately stratifying patients with AML according to the predicted prognosis: Group 1 (*n* = 22, 63%): p53(-)/MRP1(-) associated with AKR1B10(+)/AKR1B1(+) or AKR1B10(-)/AKR1B1(-); 5-year overall survival (OS), 82%–100%; Group 2 (*n* = 9, 26%): p53(-)/MRP1(-) associated with AKR1B10(+)/AKR1B1(-); 5-year OS, 68%; Group 3 (*n* = 4, 11%): p53(+) or MRP1(+); median survival, 12–14 months; 2-year OS, 0%. **Conclusions**: The Urayasu classification for AML is useful for predicting the prognosis of patients with AML. Group 1 in this classification included twice as many patients as that included in the Favorable prognosis group in the AML prognostic classification proposed by the European Leukemia Net. As the Urayasu classification for AML is based on the mechanisms of resistance to chemotherapy, it is not only useful for prognostic stratification of the patients, but also provides insights for developing more effective treatments for AML.

## 1. Introduction

The efficacy of cancer chemotherapy is determined by the tumor expression profiles of cancer-related genes and functional proteins that play important roles in the development of resistance to anticancer drugs. Resistance to anticancer therapy may appear even before the start of treatment (endogenous drug resistance) or after the start of treatment (acquired drug resistance). Drug resistance is the main cause of treatment resistance and recurrence in most malignancies. Herein, we provide a guideline for developing more effective cancer treatments and thereby obtaining better treatment outcomes based on a better understanding of the mechanisms of drug resistance [1]. We are currently engaged in studying treatment resistance caused by the expression of functional proteins involved in drug resistance in hematopoietic malignancies. We have already proposed the Urayasu prognostic classification for large B-cell lymphoma (LBCL) and aggressive T-cell lymphoma (TCL) based on the expression patterns of multiple resistance factors even prior to the start of treatment (endogenous drug resistance) [2,3]. While the prognosis of AML patients is well known to be related to gene mutations in the cancer cells [4], herein, we propose the Urayasu prognostic classification for AML based on the expression patterns of multiple resistance (endogenous) factors even prior to the start of treatment. For reference, we shall briefly outline the mechanisms of drug resistance in AML [5].

(A) Angiogenesis and vascular hyperplasia due to extracellular release of microenvironmental factors and escape from the immunosurveillance mechanism

(1) Non-immune microenvironmental factors

Factors that facilitate overcoming stress conditions such as hypoxia and hypoglycemia in the tumor microenvironment.

1. Glucose-regulated protein 94 (GRP94) [6,7]

GRP94 exists mainly in the endoplasmic reticulum and/or mitochondria. It is secreted outside the cell and regulates apoptosis, inflammation, and angiogenesis [6]. Upregulation of GRP94 has been observed in various cancers, including multiple myeloma, suggesting the clinical significance of developing treatment agents that can selectively target GRP94 [7].

2. Glucose-regulated protein 78 (GRP78) [8,9,10]

GRP78 is mainly expressed in the endoplasmic reticulum. The expression of GRP78 is known to be associated with cancer and is a potential therapeutic target as well [8]. Expression of this protein has been identified in pediatric patients with high-risk B-cell acute lymphoblastic leukemia (B-ALL) [9]. It has been reported that combined GRP78-CAR-T cell + dasatinib therapy can substantially enhance its effector function [10].

3. Transforming growth factor β1 (TGFβ1) [11,12]

TGFβ1 is involved in cell growth and differentiation, apoptosis, angiogenesis, and cellular immunity. In the early stages of cancer development, it inhibits cell transformation and prevents the progression of cancer, whereas in the later stages, it promotes tumor progression through inducing mesenchymal transition, triggering angiogenesis, and inducing immunosuppression [11]. Failure of regulation of the TGFβ1 pathway has been reported in many hematological malignancies, including myelofibrosis, AML, and malignant lymphoma [12].

Tumor necrosis factor α1 (TNFα1) [13]

TNFα is involved in the progression and relapse of AML and is associated with decreased patient survival [13]. We have previously reported that fibrosis caused by TGF-β1 and TNF-α1 produced by hematologic malignancies is associated with a poor prognosis [14]. Soluble TNF initiates TNFR1 signaling, but not TNFR2 signaling, despite receptor binding, except as the second messenger [15]. TNFR1 is expressed in AML and promotes proliferation of the tumor cells [16]. TNFR1 and Caspase10 expressed in ALL induce cell death [17].

(2) Immune microenvironmental factors (3 types)

(i) Programmed cell death-1 (PD-1) (CD279) [18]

(ii) Programmed cell death–ligand 1(PD-L1, CD274) [19]

In AML, PD-L1 expression is known to be associated with a poor prognosis.

(iii) Programmed cell death–ligand 2 (PD-L2, CD273) [20]

In AML, tumor cell surface expression of PD-1/PD-L1,2 is clinically significant, and these patients benefit from immune checkpoint inhibitor therapy.

(B) Equilibrative nucleoside transporter 1 (ENT1) decreases drug uptake [21]

In patients with AML, a decrease in cytarabine influx associated with a decrease in tumor ENT1 expression could cause treatment resistance.

(C) Enhanced drug elimination

(1) Multidrug resistance 1 (MDR1) [22]: MDR1 could be a useful molecular marker of the prognosis in AML patients.

(2) Multidrug resistance-associated protein 1 (MRP1) [23]: Tumor MRP1 expression has a significant effect on survival in patients with AML.

(3) Multidrug resistance-associated protein 4 (MRP4) [24]: MRP4 could become established as a promising novel target to develop agents inhibiting tumor growth and inducing apoptosis.

(D) Changes in drug metabolism

(1) Cytochrome P450 3A4 (CYP3A4) [25]: Combined use of FLT3 TKIs and CYP3A4 inhibitors could be a promising strategy for AML.

(2) CYP2B6 [26]: CYP2B6 variants are significantly associated with the risk level in acute leukemia patients.

(3) Aldo-keto reductase family 1 member C3 (AKR1C3) [27]: In AML and T-cell acute lymphoblastic leukemia (T-ALL), AKR1C3 expression in the tumor cell cytoplasm degrades doxorubicin hydrochloride, causing treatment resistance.

(4) AKR1B1 [28]: AKR1B1 induces tumor cell proliferation in the late stage of AML. AKR1B1 is very similar in structure to AR1B10, and the two may competitively inhibit each other’s actions.

(5) AKR1B10 [29]: The intracellular concentration of daunomycin is decreased mainly by AKR1B1 and AKR1C3; these two proteins also decrease the intracellular concentrations of idarubicin, but only by about one-fifth. AKR1B10 decreases the intracellular concentration of not only daunomycin, but also of idarubicin. AKR1B10 also catalyzes reduction in the carbonyl groups of daunomycin and idarubicin, drugs used in the treatment of AML, in the cytoplasm, converting the drugs into water-soluble inactive alcohols. Like AKR1C3, it is also involved in the development of cisplatin resistance. AKR1B10 is also considered as playing a central role in the development of cyclophosphamide resistance. On the other hand, AKR1C3 is known to be associated with the development of methotrexate as well as vincristine resistance. AKR1B10 expression is regulated by genes expressed on chromosome 7q33. In the event of missing chromosome 7, and consequent failure of AKR1B10 regulation, the protein function is enhanced. Dasatinib, a Bcr-Abl tyrosine kinase inhibitor, has a moderating effect on AKR1B10 and is expected to be applicable to the treatment of AML because it inhibits the metabolism of daunomycin and idarubicin [30]. Ibrutinib, a tyrosine kinase inhibitor, has a moderating effect on AKR1C3 and is expected to be applicable to the treatment of AML because it inhibits the metabolism of doxorubicin.

(E) Other functional proteins

(1) Thymidine phosphorylase (TP) [31]: Expression of TP, which is involved in resistance to malnutrition, angiogenesis, infiltration, and metastasis, in lymphomas, is associated with poor patient prognosis due to its anti-apoptotic and angiogenic effects.

(2) P53 [32]: After induction chemotherapy for AML, the p53 protein conformation could shift from short-chain to long-chain p53 protein, leading to treatment resistance.

(3) MYC [33]: MYC gene-related abnormalities in AML are associated with other negative prognostic factors, such as complex karyotypes and advanced age, although they are observed in only less than 1% of cases.

(4) Glutathione sulfate transferase (GST) [34]: The GST1 genotype may be useful for selecting an appropriate chemotherapy regimen for AML.

## 2. Materials and Methods

### 2.1. Patients and Sample Collection

We enrolled 35 patients with AML (excluding those with AML M3) who received standard combined idarubicin + cytarabine treatment as the initial remission induction therapy at our hospital between 2015 and 2020. In this study, we have explained that we excluded M3 FAB since it is not representative of the FAB categories. In addition to combined Cytarabine + Idamycin therapy, ATRA is the most effective treatment for AML M3. Therefore, AML M3 was excluded because the drugs used are different and treatment resistance analysis would be more complicated. The distribution of the disease types in the patients is shown in Table 1. We performed immunohistochemical (IHC) staining of formalin-fixed paraffin-embedded biopsy specimens obtained from the patients to determine the expressions of 23 proteins that have previously been reported as being treatment-resistance factors; positive and negative staining results were determined by observation under an optical microscope. Biopsies were taken from all patients prior to the start of induction chemotherapy. In this study, the analysis was limited to the period before induction chemotherapy. We then compiled and performed a retrospective analysis of the results. We used variables such as anticancer drug metabolic factors in the analysis model and compared the overall survivals (OS) of the patients after the initial remission induction therapy by the log-rank test. The 2022 ELN risk classification [35] was used as the control. The approval code is U17-0016. Date of approval by our institution’s Ethics Committee: 9 December 2022.

### 2.2. Immunohistochemistry

Tissue biopsy specimens obtained from the patients were fixed in formalin and embedded in paraffin to prepare tissue blocks, sectioned, and subjected to IHC staining. The primary antibodies, directed against the following major proteins involved in anticancer drug metabolism, were as follows: (1) GRP94: Proteintech (Rosemont, IL, USA), clone 1H10B7 (monoclonal antibody generated against the N-terminal region of full-length HSP90b1); (2) CYP3A4: Sigma-Aldrich (St. Louis, MO, USA), SAB1400064 (polyclonal antibody generated against CYP3A4); (3) AKR1C3: Proteintech, 11194-1-AP (polyclonal antibody generated against AKRC3); (4) MDR1 (P-glycoprotein): Proteintech, 22336-1-AP (polyclonal antibody generated against MDR1); (5) MRP1 (CD9): Proteintech, 60232-1-IG (monoclonal antibody generated against the N-terminal region of full-length MRP1); (6) TGF-beta1: Proteintech, 21898-1-AP (polyclonal antibody generated against TGF-beta); (7) GRP78: Proteintech, 66574-1-IG (monoclonal antibody generated against the N-terminal region of full-length GRP78); (8) glutathione S-transferase-kappa1 (GST): Proteintech, 14535-1-AP (polyclonal antibody generated against GST1); (9) thymidine phosphorylase: Abcam (Cambridge, UK), ab226917 (polyclonal antibody generated against thymidine phosphorylase); (10) MRP4 (ABCC4): SANTA CRUZ BIOTECHNOLOGY (Dallas, TX, USA), SC-376262 (monoclonal antibody generated against the N-terminal region of full-length MRP4 [amino acid 1–280]); (11) CYP2B6: LifeSpan BioSciences, Inc. (Seattle, WA, USA), LS-C352084 (polyclonal antibody generated against CYP2B6); (12) TNF1-alpha: Sigma-Aldrich, SAB4502982 (polyclonal antibody generated against TNF1-alpha); (13) PD-1: (14) PD-L1: Proteintech, 66248-1-IG, mouse IgG1 monoclonal antibody. Clone 2B11D11; (15) PD-L2 Proteintech,18251-1-AP 16, rabbit IgG polyclonal antibody; (16) P53: Cell Signaling Technology, Inc. (3 Trask Lane Danvers, MA, USA), DO-7 mouse monoclonal antibody #48818; (17) c-MYC: Abcam (Kendall Sq Cambridge, MA, USA), Y69 clone ab32072; (18) ENT-1 (equilibrative nucleoside transporter 1): Proteintech, 1337-1-AP rabbit IgG polyclonal antibody; (19) AKR1B1: Sigma-Aldrich (3050 Spruce Street Saint Louis, MO, USA), rabbit polyclonal antibody HPA052751; (20) AKR1B10:Sigma-Aldrich (3050 Spruce Street Saint Louis, MO, USA), rabbit monoclonal antibody HPA020280. After the immunostaining, two pathologists definitively determined the results of the IHC staining. The IHC staining was judged as positive when more than 50% of the tumor cells showed positive staining (weakly positive staining was also considered). The concordance rate between the two pathologists for the staining results was about 83%. In the case of disagreement between the two pathologists, the final diagnosis was arrived at by consensus.

### 2.3. Statistical Analysis

To confirm the association between the OS after the initial remission induction therapy with idarubicin plus cytarabine and poor prognostic factors/factors involved in anticancer drug metabolism, survival curves were plotted by the Kaplan–Meier method, and factors significantly associated with the OS were evaluated by the log-rank test. The significance level in the statistical tests was set at 0.05 (two-tailed) and *p* < 0.05 was considered as indicative of a statistically significant difference. Statistical analyses were performed using EZR version 2.7-1 software (Saitama Medical Center, Jichi Medical University, Saitama, Japan) [30]. Multiple comparisons were not considered because of the exploratory nature of this study.

## 3. Results

### 3.1. Kaplan–Meier Survival Curves and Comparisons Among Groups (Log Rank Test)

#### 3.1.1. Overall Survival of AML Patients with and Without Expression of a Prognostic Factor

**Figure 1:**
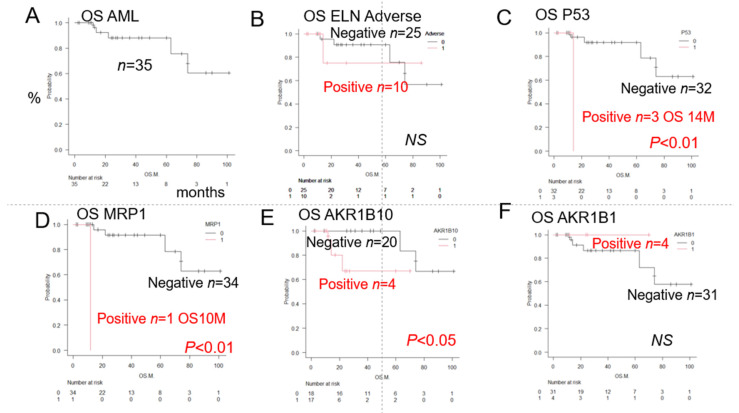
Overall survival of AML patients with and without tumor expression of the prognostic factors. Comparison of the Kaplan–Meier survival curves and disease/prognostic factors between 2 groups (log-rank test). Comparison of the overall survival (OS) after initial (3 + 7) remission induction therapy with idarubicin + cytarabine. (**A**). The median OS for AML (*n* = 35) was not reached, and the 5-year OS was 72%. Hereafter, the red letters indicate the OS in the group showing positive immunostaining of the AML blast cells for the prognostic factor, and the black letters indicate the OS in the group showing negative immunostaining for the prognostic factor. (**B**). There was no significant difference in the OS rate between the patients classified into the Adverse group (*n* = 10) and other groups (Favorable/Intermediate) according to the ELN classification; the median OS was not reached, and the 5-year OS was 72% (Not significant [NS]). (**C**). p53 positivity was associated with a significantly reduced OS (*n* = 3; median OS 14 months, 2-year OS 0%, *p* < 0.01). (**D**). MRP1 positivity was associated with a significantly reduced OS (*n* = 1; OS 12 months; 5-year OS 72%, *p* < 0.01). (**E**). AKR1B10 positivity was associated with a significantly reduced OS (*n* = 17; median OS not reached; 5-year OS 63%, *p* < 0.05). (**F**). AKR1B1 positivity had no significant effect on the OS, but it probably inhibited AKR1B10 and enhanced the effect of idarubicin, resulting in a very good prognosis (*n* = 4; median OS not reached; 2-year OS 72%, NS).

As shown in Figure 1, the median OS was not reached in the 35 patients after the initial remission induction therapy. The 5-year OS was 72%. A log-rank test was performed to compare the OS rates in the patients in relation to the expression patterns of factors involved in anticancer drug metabolism. There were no significant differences in the OS rates among the patients included in the Adverse group according to the European Leukemia Net (ELN) classification. However, the patients in this group showed significant differences in the expression patterns of 3 factors, namely, p53, MRP1, and AKR1B10. Furthermore, there were no significant differences in the expression rate of AKR1B1, which competitively inhibits the metabolic degradation of idarubicin by AKR1B10; potentially, inhibition of AKR1B10 by AKR1B1 results in reduced metabolic degradation and enhanced effect of idarubicin, which results in a relatively good prognosis.

#### 3.1.2. Overall Survival of AML Patients with and Without Expression of the Two Prognostic Factors

**Figure 2:**
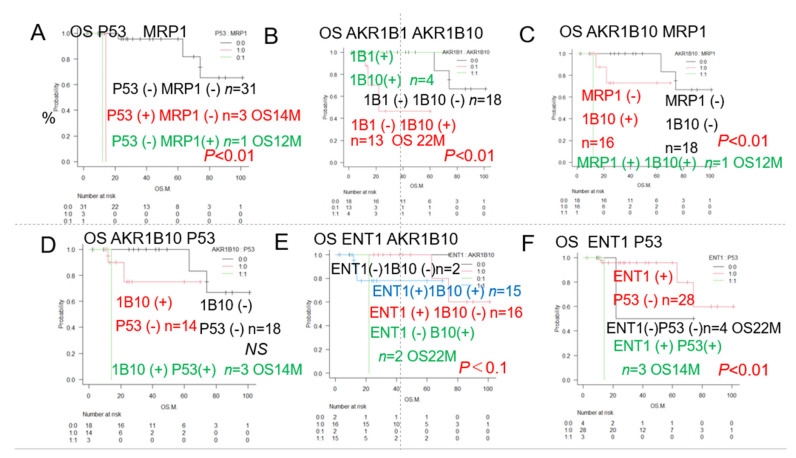
Overall survival (OS) of AML patients with and without expressions of the prognostic factors. Comparison of the Kaplan–Meier survival curves and prognostic factors, positive/negative immunostaining between 2 groups (log-rank test). The combinations of the two groups are indicated in black texts and black lines when both groups are negative, and in red, green, and blue when they are not, respectively. See Table 2 for the correlation between p53 and other factors. (**A**). A markedly significant difference was observed for different combinations of P53 and MRP1 expression (*p* < 0.01); p53 (-) MRP1 (+): *n* = 1, OS 12 months, 2-year OS 0%; p53 (+) MRP1 (-): *n* = 3, OS 14 months, 2-year OS 0%; p53 (-) MRP1 (-): *n* = 31, median OS not reached, 5-year OS 82%. (**B**). A significant difference was observed between different combinations of AKR1B10 and AKR1B1 expression (*p* < 0.01; AKR1B1 (-) AKR1B10 (+): *n* = 13, OS 22 months, 5-year OS 43%; AKR1B1 (-) AKR1B10 (-): *n* = 18, OS not reached, 5-year OS 82%. (**C**). Markedly significant differences were observed for different combinations of MRP1 and AKR1B10 expression (*p* < 0.01); MRP1 (+) AKR1B10 (+): *n* = 1, OS 12 months, 2-year OS 0%; MRP1 (-) AKR1B10 (+): *n* = 16, median OS not reached, 5-year OS 56%; MRP1 (-) AKR1B10 (-): *n* = 18, median OS not reached, 5-year OS 82%. (**D**). No significant difference was observed for different combinations of AKR1B10 and p53 expression; the association was observed (NS). AKR1B10 (+) p53 (+): *n* = 3, OS 14 months, 2-year OS 0%; AKR1B10 (+) p53 (-): *n* = 14, median OS not reached, 5-year OS 74%. AKR1B10 (-) p53 (-): *n* = 18, median OS not reached, 5-year OS 82%. (**E**). No significant difference was observed for different combinations of ENT1 and AKR1B10 expression; the association was observed (NS). ENT1 (-) AKR1B10 (+): *n* = 2, OS 22 months, 2-year OS 0%; ENT1 (+) AKR1B10 (+): *n* = 15, median OS not reached, 5-year OS 78%; ENT1 (+) AKR1B10 (-): *n* = 16, median OS not reached, 5-year OS 100%; ENT1 (-) AKR1B10 (-): *n* = 2, median OS not reached, 5-year OS 100%. (**F**). Markedly significant differences were observed for different combinations of ENT1 and AKR1B10 expression (*p* < 0.01); ENT1 (+) p53 (+): *n* = 3, OS 14 months, 2-year OS 0%; ENT1 (-) p53 (-): *n* = 4, OS 22 months, 5-year OS 52%; NT1 (-) p53 (-): *n* = 28, median OS not reached, 5-year OS 82%; ENT1 (-) p53 (-): *n* = 4, median OS not reached, 5-year OS 100%.

As shown in Figure 2, the results of the univariate analysis showed that the decrease in OS rates differed significantly among patients showing different patterns of expression of p53, MPR1, AKR1B10, and AKR1B1 (a competitive inhibitor of AKR1B10). We added the expression of ENT1, a pump that promotes the influx of cytarabine into the blast cells in AML, to these factors, and based on the patterns of expression, we performed a stratified analysis of the OS rates. The results showed significant differences in the OS rates among patients, showing different combinations of p53 and MRP1, AKR1B1 and AKR1B10, AKR1B10, and MRP1, as well as p53 and ENT1 expressions. We expected significant impacts of expression of the idarubicin-metabolizing enzyme AKR1B10 (poor prognosis) and cytarabine influx pump ENT1 (good prognosis) used in remission induction therapy, but no significant differences in the OS related to the expressions of these proteins were observed, although their expressions tended to be associated with a good prognosis.

The results of the univariate analysis showed that the decrease in OS rates differed significantly among patients showing different patterns of expression of p53, MPR1, AKR1B10, and AKR1B1 (a competitive inhibitor of AKR1B10).

The results of univariate analysis showed that the decrease in OS rates differed significantly among patients who showed many different patterns of expression.

**Figure 3:**
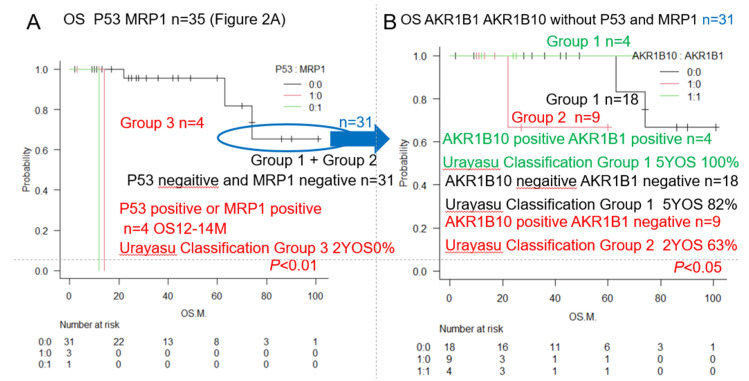
Overall survival (OS) of AML patients with and without expressions of the prognostic factors (AML Urayasu classification). Kaplan–Meier survival curves and comparisons of the survival rates among the three groups (Groups 1–3) (log-rank test) (**A**). Urayasu classification for AML (first step). Immunostaining for p53 and MRP1 was performed in all the 35 cases enrolled in this study. Group 3: The median OS in the four cases that were p53(+) and/or MRP1(+) was 12–14 months (*p* < 0.01), indicating an extremely poor prognosis. The median OS was not reached in the remaining 31 cases (Group 1 + Group 2), which were all p53-negative and MRP1-negative and showed a 5-year survival rate of 82% (*p* < 0.01), indicative of a good prognosis. (**B**). Urayasu classification for AML (second step). Immunostaining for AKR1B1 and AKR1B10 was performed in the 31 cases of Group 1 + Group 2, who were p53-negative and MRP1-negative. Group 1 consisted of 22 patients, including four patients who were AKR1B10-positive/AKR1B1-positive (5-year OS 100%) and 18 patients who were AKR1B10-negative/AKR1B1-negative (5-year OS 82%). Group 2 consisted of nine patients who were AKR1B10-positive/AKR1B1-negative (2-year OS 63%).

Table 2 shows a summary of the results of the analysis of the expression (IHC staining) patterns of the 23 different proteins/antibodies. Survival rates assessed by the Kaplan–Meier method, the median cumulative survival rate, 95% confidence interval (CI), as well as the results of comparison among groups (*p*-value: log rank test) are shown. Poor prognostic factors were identified based on the differences in survival. Significant (*p* < 0.05) poor prognostic factors are marked with (#). The classification of patients into the Favorable, Intermediate, and Adverse groups (Figure 1B) by the conventional prognostic factors as per the ELN classification, as well as by complex karyotypes and del (7) showed a trend towards correlation with the OS, but no significant differences were observed. However, tumor expressions of p53, MRP1, and/or AKR1B10 were significantly associated with decreased OS rates, as shown in Figure 1. Conversely, a trend toward improved OS was observed in patients showing tumor AKR1B1 (competitive inhibitor of AKR1B10) expression, although the difference was again not statistically significant. Of the combinations of two factors that showed the most significant associations with the OS, at least 11 combinations included p53. Of these, the combination of p53 and MRP1, in particular, showed the strongest association with the OS (Figure 2A); in addition, the combination of AKR1B10 and ALR1B1 A was also significantly associated with the OS (Figure 2B). Therefore, we focused on the patterns of expressions of 4 factors, namely, p53, MRP1, AKR1B10, and AKR1B1, and devised the Urayasu classification for AML, a prognostic classification based on the mechanisms of development of drug resistance in AML, as shown in Figure 3.

### 3.2. AML Urayasu Classification

As shown in Figure 3, to devise the Urayasu classification for AML based on the expression patterns of important treatment resistance factors, in addition to the status of expressions of p53 and MRP1, we proposed the addition of the status of expressions of AKR1B10, an enzyme that degrades idarubicin, and AKR1B1, a competitive inhibitor of AKR1B10. Specifically, based on the results shown in Figure 2A, we classified patients showing p53 positivity and/or MRP1 positivity (*n* = 4) as Group 3 or the poor prognosis group. Patients showing negative tumor expressions of p53 and MRP1 (*n* = 31) were classified into two groups (Group 1 or Group 2) with a more favorable prognosis than Group 3. Group 2 consisted of patients who were AKR1B10-positive, but AKR1B1-negative (*n* = 9), and Group 1 consisted of patients with the other possible combinations of AKR1B10/AKR1B1 expressions (AKR1B10-positive/AKR1B1-positive or AKR1B10-negative/AKR1B1-negative, AKR1B10-negative/AKR1B1-positive). Significant differences in the OS were observed among the three groups. These results are also shown in Table 2.

**Figure 4:**
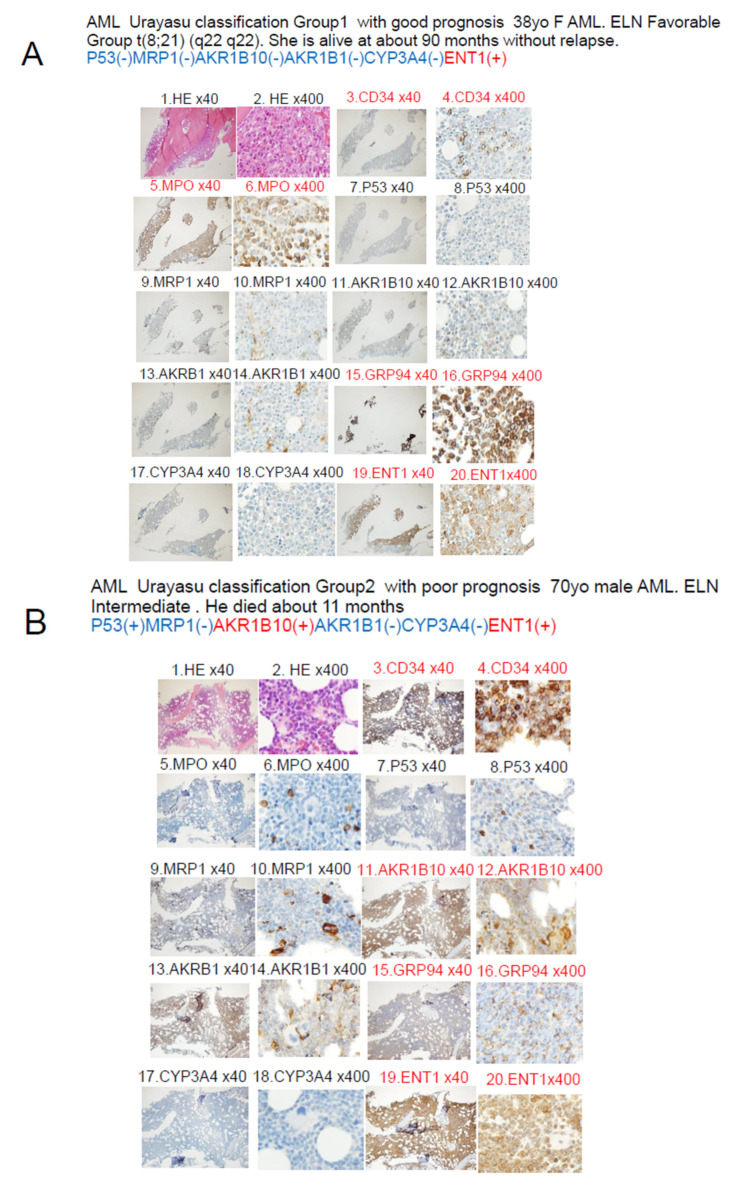

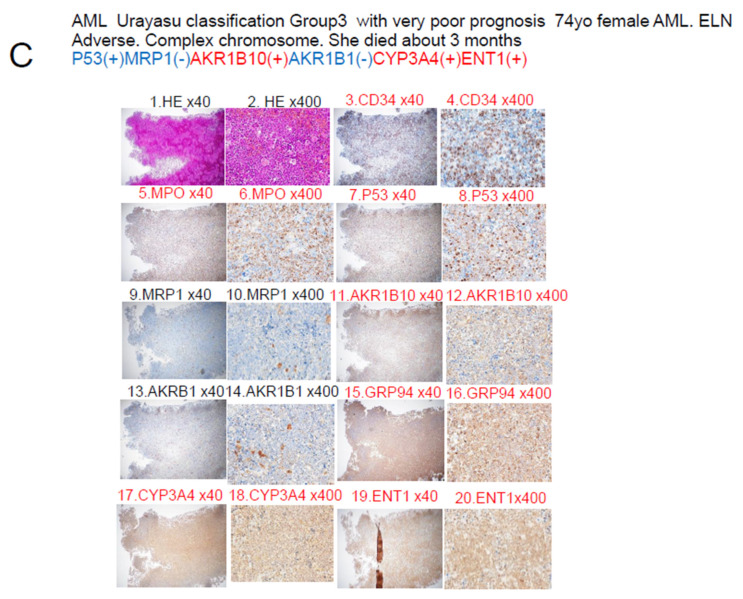
(**A**). The results of immunohistochemical staining of the bone marrow at disease onset in a representative patient classified into Group 3 of the Urayasu classification for AML. Representative example of Group 1. The 38-year-old female patient with the t(8;21)(q22;q22.1) chromosomal abnormality. She was classified into the Favorable prognosis group by the ENL prognostic classification. The patient has maintained remission after induction therapy with cytarabine plus idarubicin. High-dose cytarabine was repeated thrice for consolidation. The patient has been in long-term first complete remission for 90 months. The results of immunohistochemical staining were as follows: P53(-)MRP1(-)AKR1B10(-)AKR1B1(-)CYP3A4(-)ENT1(+); remission induction therapy with cytarabine plus idarubicin, and high-dose cytarabine consolidation therapy were effective. (**B**). The results of immunohistochemical staining of the bone marrow at disease onset in a representative patient classified into Group 2 of the AML Urayasu classification. The red font indicates positivity, and the black font indicates negativity. HE is an abbreviation for hematoxylin–eosin stain. Representative example of Group 2. 70-year-old male patient with AML. Classified in the Intermediate prognosis group by ELN. There was a t(2;11) 11q23 chromosomal abnormality. First-line remission induction therapy with cytarabine plus idarubicin was ineffective, and second-line induction therapy with azacytidine plus venetoclax also proved ineffective. The immunohistochemical staining results were as follows: P53(+)MRP1(-)AKR1B10(+)AKR1B1(-)CYP3A4(-)ENT1(+). The treatment resistance was considered attributable to expressions of the tumor growth factor, P53, and of the idamycin-metabolizing enzyme, AKR1B10. (**C**). The results of immunohistochemical staining of the bone marrow at disease onset in a representative patient classified into Group 3 of the AML Urayasu classification. The red font indicates positivity, and the black font indicates negativity. HE is an abbreviation for hematoxylin–eosin stain. The patient was a 74-year-old female who was diagnosed as having a complex-karyotype AML and received combined idarubicin + cytarabine treatment as the initial remission-induction therapy, but CR was not achieved. As re-induction therapy, she received combined venetoclax + azacitidine treatment, but CR was not achieved, and the patient died three months later. Consent for autopsy could not be obtained. The patient showed tumor p53-positivity and was classified into Group 3. The tumor was MRP1-negative, AKR1B10-positive, and AKR1B1-negative. Her disease was resistant to the combined treatment with idarubicin + cytarabine as the initial remission-induction therapy. A possible reason for this is that her tumor cells were p53-positive and AKR1B10-positive/AKR1B1 (a competitive inhibitor of AKR1B10)-negative, which could have led to rapid metabolism of idarubicin within the AML blast cells, which was therefore no longer able to suppress tumor cell growth. The patient received combined venetoclax + azacitidine treatment as re-induction therapy for remission, but remission was not achieved. The reason for this may be that the venetoclax was rapidly metabolized within the AML blast cells due to an increase in the expression of CYP3A4, which metabolizes venetoclax.

Figure 4 illustrates a case that is thought-provoking with regard to the AML Urayasu classification established on the basis of the results of IHC staining.

HE-stained slides 1 and 2 show numerous blast cells at 40× and 400× magnification. Slides 3 and 4 show positive staining of several blast cells for CD34, suggesting the diagnosis of acute leukemia. Slides 5 and 6 show positive results for myeloperoxidase (MPO) staining, and the patient was diagnosed as having AML. Slides 7 and 8 show positive staining for p53, and the patient was classified into Group 3 of the Urayasu classification for AML, a group with an extremely poor prognosis. Slides 9 and 10 showed negative staining of the tumor cells for MRP1. Slides 11 and 12 show positive staining of the tumor cells for AKR1B10, indicating the likelihood of idarubicin having been rapidly metabolized within the blast cells and thereby showing reduced efficacy. Furthermore, slides 13 and 14 show negative staining of the tumor cells for AKR1B1, which competitively inhibits AKR1B10, which explains why the patient remained refractory to treatment. Slides 15 and 16 show positive staining of the tumor cells for GRP94, which is expressed in many AML cells, which are known to confer the tumor cells with the ability to adapt to the microenvironment. Slides 17 and 18 show positive staining of the tumor cells for CYP3A4, which is involved in the metabolism of idarubicin, cytarabine, as well as venetoclax. Slides 19 and 20 show positive staining of the tumor cells for ENT-1, which functions as an intracellular influx pump for cytarabine.

**Figure 5:**
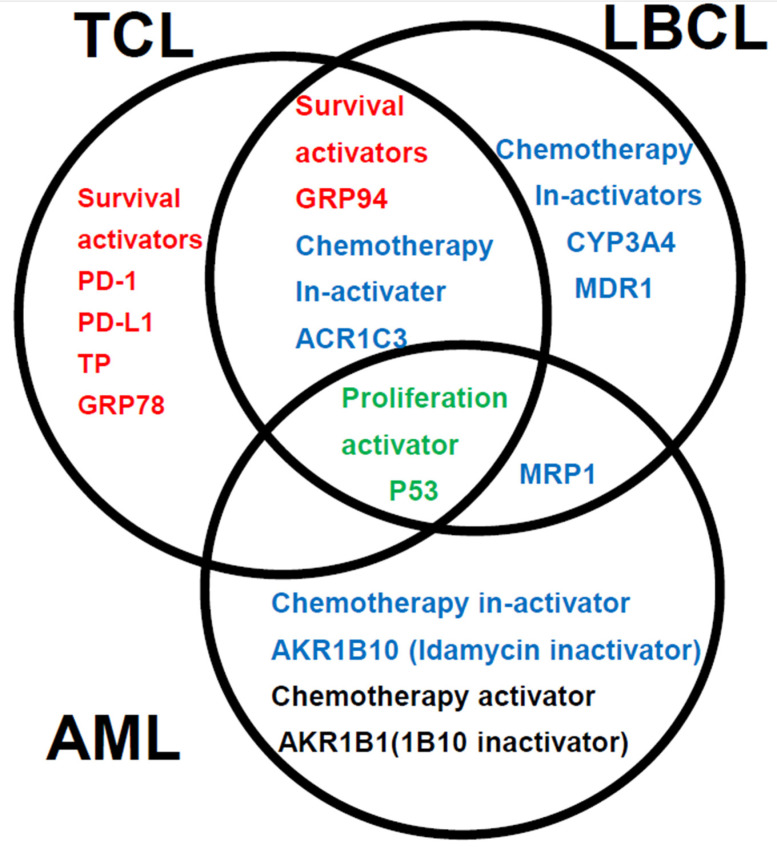
A summary of the prognostic factors associated with treatment resistance in LBCL [2], TCL Urayasu [2], and AML (this article) in an easy-to-understand Venn diagram with three circles.

Figure 5 shows a summary of the prognostic factors associated with treatment resistance in LBCL [2], TCL [3], and AML (this article) in an easy-to-understand Venn diagram with three circles. The poor prognostic factor common to all three diseases is tumor expression of the proliferation activator p53. The expressions of the microenvironmental factor, GRP94, and idarubicin-detoxifying enzyme AKR1C3 are common poor prognostic factors in TCL and LBCL. In AML and LBCL, the shared poor prognostic factor is tumor expression of the idarubicin extracellular efflux pump, MRP1. In TCL, microenvironmental factors, as well as five types of survival activators (PD-1, PD-L1, TP, GRP78, and GRP94), have also been identified as important prognostic factors. In LBCL, CYP3A4, which detoxifies doxorubicin, and the doxorubicin efflux pump, MDR1, are important prognostic factors. In AML, tumor expressions of AKR1B10, which degrades idarubicin, and AKR1B1, which inhibits the metabolism of idarubicin are important prognostic factors.

Table 3 shows a comparison of the Urayasu classification for AML and the 2022 ELN risk classification.

Our analysis of the OS yielded slightly better results; this was thought to be because a higher proportion of patients was classified into the Favorable/Intermediate prognosis groups, and a lower proportion into the Adverse prognosis group by the Urayasu classification for AML, so it was easier to extract groups with a good prognosis by this classification approach.

## 4. Discussion

In general, IHC staining with visualization under an optical microscope is considered as very useful test, as it allows confirmation of the positivity/negativity while also allowing the tumor cells to be identified. The result of IHC staining is rated as weakly positive when 50% or more of the tumor cells show positive staining. In this study, we performed IHC staining of biopsy specimens obtained from AML patients for 23 treatment resistance factors [6,7,8,9,10,11,12,13,14,15,16,17,18,19,20,21,22,23,24,25,26,27,28,29,30,31,32,33,34] selected from a review of the literature. Based on the results, we have proposed a new prognostic classification (Group 1 to Group 3), terming it as the Urayasu classification for AML. We believe that this classification would be useful to predict the efficacy of the initial 3 + 7 remission induction therapy of idarubicin + cytarabine in patients with new-onset AML. In this study, we classified the 35 study participants according to the proposed Urayasu classification for AML as follows, based on the results shown in Figure 3 and Table 2: (1) Group 1 (*n* = 22; Favorable prognosis): Of the 35 patients, 22 (63%) showed the following IHC staining patterns: p53(-)/MRP1(-), and AKR1B10(+)/AKR1B1(+) or AKR1B10(-)/AKR1B1(-). The 5-year OS of the patients in this group was 82%-100% (In the ELN classification for AML [35], which is the conventional prognostic classification, *n* = 562, 37%, 59%, 5-year OS: approximately 50%, median OS: 4 years); (2) Group 2 (*n* = 9; Intermediate prognosis): the nine patients (26%) of this group showed the following IHC staining pattern: p53(-)/MRP1(-) and AKR1B10(+)/AKR1B1(-); the 5-year OS was 68% (ENL classification, *n* = 355, 23%, 5-year OS: approximately 20%, median OS: approximately 15 months); Group 3 (*n* = 4; Adverse prognosis): the four patients (11%) of this group showed the following IHC staining pattern: p53(+) and/or MRP1(+); the median survival was 12–14 months, and the 2-year OS was 0% (ENL classification, *n* = 616, approximately 40%, 5-year OS: 8%, median OS: approximately 15 months). Even though the Urayasu classification for AML is based on the IHC staining results obtained from a relatively small number of patients, we believe that it will contribute to the stratification of the treatment and advances in treatment methods for patients with AML, as it is based on the expression patterns of treatment resistance factors in the tumor cells. The sample size was too small to draw any definitive conclusions. In this AML analysis, as shown in Table 2, there was no significant difference in the OS as compared with the ELN classification. A multivariate analysis showed a significant difference in relation to the AKR1B10 expression, with an odds ratio of 0.872 (*p* < 0.05). In other words, the OS was significantly lower in cases showing positive tumor expression of AKR1B10. We have also stated, however, that there may be problems with multicollinearity due to vif > 5, and added that there could be potential selection bias and influence of factors such as the age and presence of comorbidities.

Table 3 shows a comparison of the AML Urayasu classification and the 2022 ELN risk classification. A multivariate analysis showed a significant difference in relation to the AKR1B10 expression, with an odds ratio of 0.872 (*p* < 0.05). In other words, the OS was significantly lower in cases showing positive tumor expression of AKR1B10. We have also stated, however, that there may be problems with multicollinearity due to vif > 5, and added that there could be potential selection bias and influence of factors such as the age and presence of comorbidities.

Our analysis of the OS yielded slightly better results; this was thought to be because a higher proportion of patients was classified into the Favorable/Intermediate prognosis groups and a lower proportion into the Adverse prognosis group by the Urayasu classification for AML as compared with the 2022 ELN risk classification, making it easier to extract groups with a good prognosis. The Urayasu classification for AML was able to identify a higher proportion of patients with a favorable prognosis, who may not necessarily require allogeneic transplantation, than the ELN classification. Furthermore, analysis of the expression patterns of treatment resistance factors as the basis for this classification would aid in the selection of future treatment methods for the patients. For example, in patients classified into Group 3, that is, those showing tumor p53 positivity, it is difficult to expect remission with chemotherapy alone [32], and in patients with BCL2 positivity and CYP3A4 (venetoclax-metabolic enzyme) negativity, venetoclax should be used [36]. In patients showing MCL1 positivity, the use of azacitidine should be considered [37]. Figure 4 illustrates a case that is thought-provoking with regard to the Urayasu classification for AML. The patient showed tumor p53 positivity and was classified into Group 3; she was MRP1 [23] (doxorubicin efflux pump)-negative, AKR1B10-positive, and AKR1B1-negative. Her disease was resistant to the initial remission induction therapy with idarubicin + cytarabine. This could be attributable to loss of the tumor suppressor function due to p53 positivity [32], as well as to idarubicin being rapidly metabolized within the AML blast cells due to the AKR1B10-positive/AKR1B1-negative staining result [29]. The patient was treated with venetoclax + azacitidine as second-line induction therapy [38], but this treatment also failed to induce remission. This could have been due to tumor expression of CYP3A4 causing rapid metabolism of venetoclax within the AML blast cells [36]. The patient died within 3 months of the diagnosis. In the future, it may be possible to devise individualized AKR1B10 inhibitor therapy in patients with AML. AKR1B10 shows 70.6% amino acid sequence homology with aldose reductase AKR1B1, and the structures and substrate specificities are also extremely similar. Since many compounds that inhibit AKR1B10 also inhibit AKR1B1 to a similar extent, compounds that selectively inhibit each enzyme have been sought and generated [39]. HCCFA, 7-hydroxy-2-(4-methoxyphenylimino)-2H-chromene-3-carboxylic acid benzylamide (HMPC) generated through such a process strongly and selectively inhibits AKR1B10 [39]. AKR1B10 is also involved in cisplatin resistance. Furthermore, it is also considered to cause strong resistance to cyclophosphamide, and there are concerns that the cyclophosphamide effect in cyclophosphamide + total body irradiation or cyclophosphamide + busulfan regimen used for pre-allogenic transplantation conditioning could be compromised with increase in the relapse rate in patients with tumor expression of AKR1B10 [39]. In addition, AKR1B10 is regulated by genes on chromosome 7q33. In cases with absent chromosome 7, AKR1B10 regulation would fail, resulting in activation of the enzyme. The Bcr-Abl tyrosine kinase inhibitor dasatinib has a moderating effect on AKR1B10 and is expected to be applicable to the treatment of AML, because it inhibits the metabolism of daunomycin or idurubicin [29,40]. NSAIDs such as N-phenyl-anthranilic acid, diclofenac, and glycyrrhetinic acid competitively inhibit AKR1B10 [41]. Based on the above, inhibitors such as HCCFA, dasatinib, and NSAIDs are considered promising for use as AKR1B10 inhibitors in patients with AML. There are reports of MRP1 expression in some cases of normal-karyotype AML [23,42]. The patient with MRP1 positivity in this case series had Inversion 16+ 8 trisomy. Her disease relapsed about 11 months after diagnosis, and she became treatment-resistant. MRP1 is located on chromosome 16 at p13.13-p13.12. The tumor cells in cases of AML with inversion of chromosome 16, inv(16)(p13q22) often lack MRP1 gene expression, resulting in a good response to chemotherapy and a good prognosis [43]. However, in our case, MRP1 was unexpectedly expressed, causing treatment resistance. The relationships among the treatment resistance factors included in the Urayasu classifications for AML, TCL [3], and LBCL [2] are summarized in Figure 5. As shown in this figure, of the poor prognostic factors, tumor p53 [32] expression is shared by all three diseases. The tumor positivity rate for p53 is high in the order of TCL (10/16, 67%), LBCL (15/42, 36%), and AML (5/35, 9%). In other words, the poor prognosis in patients with TCL is largely because of the expression of p53. p53 is an important tumor suppressor, and loss of p53 function due to mutation or other causes can lead to cancer development/progression. p53 mutation is noted in over 50% of human cancers. However, there are currently no drugs approved for the clinical treatment of cancers harboring p53 mutations [44]. The commonly expressed treatment resistance/poor prognostic factors between TCL and LBCL include GRP94 and AKR1C3. The positivity rate for the microenvironmental factor GRP94 is high in the order of AML (33/35, 94%), LBCL (38/42, 90%), and TCL (10/16, 67%). Tumor cells in AML and LBCL, as well as TCL, often use GRP94 to adapt to their environment, which may contribute to the development of treatment resistance and recurrence. The positivity rate for the enzyme AKR1C3, which inactivates doxorubicin, cyclophosphamide, and vincristine, is high in the order of LBCL (26/42, 62%), AML (17/35, 46%), and TCL (6/16, 38%). Thus, in LBCL, for which R-CHOP therapy and Pola-RCHP therapy are standard treatments, AKR1C3 could contribute to acquired treatment resistance. PAML showing tumor expression of p53 (3/35, 9%) or MRP1 (1/35, 3%) carries a poor prognosis, and although the positivity rate for both is also low at 12%, an MRP1 inhibitor has been developed [45,46]. In addition to microenvironmental factors that have a significant impact on the prognosis of TCL, AML cells can become resistant to treatment when they express the enzyme AKR1B10, which metabolizes the anthracycline-based anticancer drug idarubicin [29]. AKR1B1 shows 70.6% amino acid sequence homology with AKR1B10, and the structures and substrate specificities are also extremely similar. Since many compounds that inhibit AKR1B10 also competitively inhibit AKR1B1 to a similar extent, reducing AKR1B10 activity would eliminate treatment resistance [39]. The Abl tyrosine kinase inhibitor dasatinib has a moderating effect on the activity of AKR1B10, and is expected to be applicable to the treatment of AML because it inhibits the metabolism of daunomycin and idurubicin [29]. AKR1B10 is also known to play a central role in cisplatin resistance and cyclophosphamide resistance [29]. In fact, it must be carefully borne in mind that AKR1B10 positivity in AML could compromise the efficacy of high-dose cyclophosphamide, which forms a part of the conditioning regimen used for allogeneic hematopoietic stem cell transplantation. AKR1B10 expression is regulated by genes on chromosome 7q33. In the absence of chromosome 7, failure of regulation of AKR1B10 could lead to its activation. As shown in Table 2, we investigated the impact of deletion of chromosome 7 and AKR1B10 on the OS in our study population and found that 6 out of the 35 patients showed both deletion of chromosome 7 and AKR1B10 positivity and tended to show an extremely poor prognosis. On the other hand, in TCL, the tumor cells show excellent microenvironmental adaptability, and in LBCL, the tumor cells are considered to have an even better anticancer drug detoxification ability.

## 5. Conclusions

In summary, based on the results of the IHC analysis of tumor specimens for at least four treatment resistance proteins (p53, MRP1, AKR1B10, and AKR1B1), patients with AML can be classified into three prognostic groups, the so-called Urayasu classification for AML, as follows: Group 1 (favorable prognosis); Group 2 (intermediate prognosis); and Group 3 (poor prognosis). In the future, we plan to use MRP1 and AKR1B10 inhibitors as a stratified treatment approach based on the Urayasu classification to obtain improved treatment outcomes in patients with new-onset AML. Furthermore, we also propose to conduct similar analyses in a larger number of cases, as well as in patients with other hematological malignancies and summarize the results. IHC staining for treatment resistance proteins provides quick results and is simple and inexpensive. Furthermore, it is noteworthy that our Urayasu classification for AML classified approximately twice as many patients into the favorable prognosis group as the ELN classification. We would like to conduct further validation by analyzing a larger number of cases in the future.

## Figures and Tables

**Table 1:** Characteristics of the patients included in this analysis (*n* = 35).

Items	Count
Age > 60 years (%)	13 (37%)
Male (%)	20 (57%)
Chromosome	
CBF t(8;21) (q22;q22) *n* = 7, inv(16) (p12q22) *n* = 1	8 (23%)
Normal	10 (29%)
Complex chromosome	8 (23%)
Chromosome 7 deletion	9 (26%)
European Leukemia Net	
Favorable	12 (34%)
Intermediate	13 (37%)
Adverse	10 (29%)
Induction chemotherapy	
Idarubicin + cytarabine	35 (100%)
Outcome	
CR	27 (77%)
Non-relapse	18 (51%)
Relapse	9 (26%)
PD	8 (23%)
Allogeneic transplantation	10 (29%)

Notes: CBF: core binding factor; CR: complete remission; PD: progressive disease.

**Table 2:** The results of the univariate analysis showed that the decrease in OS rates differed significantly among patients showing different patterns of expression of the prognostic factors.

Category	Factors (♯ Significant Difference:)	*n*	Median OS (Months)	Years (Y) Survival Rate	*p* Value	Figure
Total	AML	35	NR	5Y 73%		1A
ELN	ELN Favorable group	12	NR	5Y 93%	NS	
	ELN Intermediate group	13	NR	5Y76%	NS	
	ELN Adverse group	10	NR	5Y72%	NS	1B
Chromosome abnormality	Deletion chromosome 7	9	NR	5Y78%	NS	
	Complex chromosome	8	NR	5Y73%	NS	
		12	74	5Y62%	NS	
ER stress proteins	GRP94	33	NR	5Y73%	NS	
	TGFβ1	27	63	5Y90%	NS	
	GRP78	30	NR	5Y90%	NS	
	TNFα1	20	NR	5Y82%	NS	
OH metabolic enzyme	AKR1C3	16	NR	5Y68%	NS	
	AKR1B1	4	NR	5Y72%	NS	1F
	AKR1B10 (♯)	17	NR	5Y63%	* *p* < 0.05	1E
C metabolic enzyme	CYP2B6	0				
CHOP metabolic enzyme	CYP3A4	5	NR	3Y73%		
OH efflux pump	MDR1	5	NR	3Y72%		
	MRP1 (♯)	1	12	1Y0%	* *p* < 0.01	1D
MTX efflux pump	MRP4	0			NS	
Immune checkpoint	PD-1	0			NS	
	PD-L1	1	NR	5Y100%	NS	
	PD-L2	20	74	5Y74%	NS	
Others	TP	4	74	5Y100%	NS	
	p53 (♯)	3	14	2Y0%	* *p* < 0.01	1C
	GST	28	NR	5Y88%	NS	
	MYC	28	74	5Y82%	NS	
	ENT-1	31	NR	5Y88%	NS	
	Fibrosis (Silver stain positive)	11	NR	5Y74%	*p* > 0.05	
	BCL2	28	74	5Y84%	*p* > 0.05	
	MCL1	16	NR	5Y88%	*p* > 0.05	
Significant combination						
Urayasu classification G3	P53(+) or MRP1(+) (♯)	4	13	2Y0%	** *p* < 0.01	2A, 3, 5
Urayasu classification G2	P53(-) MRP1(-) AKR1B10(+) 1B1(-) (♯)	9	NR	2Y63%	* *p* < 0.05	3, 5
Urayasu classification G1	P53(-) MRP1(-) AKR1B10(-) 1B1(-) (♯)	22	NR	5Y82%	* *p* < 0.05	3, 5
Urayasu classification G1	P53(-) MRP1(-) AKR1B10(+) 1B1(+) (♯)	3	NR	5Y100%	* *p* < 0.05	3, 5
	P53□ENT1 (♯) P53(+) ENT1(+)□	3	14	2Y 0%	** *p* < 0.01	2F
	MRP1 ENT1 (♯) MRP1(+) ENT1(+)	1	12	2Y 0%	** *p* < 0.01	
	AKR1B10, AKR1B1 (♯) 1B10(+) 1B1(-)	13	22	5Y 44%	** *p* < 0.01	2B
	MRP1, AKR1B10□(♯) MRP1(+) 1B10(+)	1	12	2Y 0%	** *p* < 0.01	2C
	P53, BCL2 (♯) P53(+) BCL2(+)	3	14	2Y 0%	* *p* < 0.05	
	P53, MCL1 (♯) P53(+) MCL1(+)	3	14	2Y0%	** *p* < 0.01	
	P53, PD-L1 (♯) P53(+) PD-L1(-)	2	14	2Y0%	** *p* < 0.01	
	P53, PD-L2 (♯) P53(+) PD-L2 (+)	3	14	2Y0%	* *p* < 0.05	
	P53, CYP3A4 (♯) P53(+) CYP3A4(+)	1	NR	NR	** *p* < 0.01	
	P53, GRP78 (♯) P53(+) GRP78(+)	3	14	2Y0%	** *p* < 0.01	
	P53, GRP94 (♯) P53,(+) GRP94(+)	3	14	2Y0%	** *p* < 0.01	
	P53, AKR1C3 (♯) P53(+) AKR1C3(+)	2	14	2Y0%	** *p* < 0.01	
	P53, TGF beta1 (♯) P53(+) TGF beta1(+)	3	14	2Y0%	* *p* < 0.05	
	P53, MYC (♯) P53(+) MYC(+)	3	14	2Y0%	* *p* < 0.05	
	P53, GST (♯) P53(+) GST(+)	1	NR	NR	* *p* < 0.05	
Combinations with a tendancy towards association with the OS						
	Del 7, AKRB10(♯) Del 7(+) 1B10(+)	6	14	2Y 0%	NS	
	BCL2, MCL1	23			NS	
	AKR1B10, P53	35			NS	
	ENT1, AKR1B10				NS	2E
	P53, AKR1B10 P53(+) AKR1B10(+)	3	14	2Y 0%	NS	2D

Notes: OS: overall survival; AML: acute myeloid leukemia; NR: not reached; ELN: European Leukemia Net; ER: endoplasmic reticulum; GRP94: glucose regulated protein 94; TGFβ1: transforming growth factor β1; OH: oncovin hydroxyl doxorubicin; GRP78: glucose regulated protein 78;TNFα1: tumor necrosis factor α1; AKR1C3: aldo-keto reductase family 1 member C3; AKR1B1: aldo-keto reductase family 1 member B1; AKR1B10; aldo-keto reductase family 1 member B10; C: cyclophosphamide; CYP2B6: cytochrome P450 2B6; CYP3A4: cytochrome P450; MDR1: multidrug resistance; MRP1 multidrug resistance-associated protein 1; MTX: methotrexate; PD-1: programmed cell death-1; PD-L1: programmed cell death–ligand 1; TP: thymidine phosphorylase; GST: glutathione sulfate transferase; ENT1: equilibrative nucleoside transporter 1; BCL2: B-cell/CLL lymphoma 2; MCL1; myeloid cell leukemia sequence 1; Del: deletion. ♯ mean Significant difference * means *p* < 0.05 significance. ** means *p* < 0.01 significance.

**Table 3:** Comparison of the Urayasu classification for AML and 2022 ELN risk classification.

Classification	Group 1 (Favorable)	Group 2 (Intermediate)	Group 3 (Adverse)	Figure
AML Urayasu	P53(-)MRP(-)AKR1B10(+)	P53(-)MRP(-)AKR1B10(+)	P53(+) or MRP1(+)	1C–F
Classification	AKR1B1(+) or	AKR1B1(-)	□	2AB, 3AB
□	P53(-)MRP(-)AKR1B10(-)	□	□	□
□	AKR1B1(-)	□	□	□
□	Cases *n* = 22 (63%)	Cases *n* = 9 (26%)	Cases *n* = 4 (11%)	□
□	OS 5 Y 82%-100%	OS 2 Y 63%	OS 2 Y 0%	□
□	Median OS NR	Median OS NR	Median OS 12–14 M	□
ELN AML risk	Cases *n* = 562 (37%)	Cases *n* = 355 (23%)	Cases *n* = 616 (40%)	1B
Classification	OS 5 Y 50%	OS 5 Y 20%	OS 5 Y 8%	□
□	Median OS 4 Y	Median OS 15 M	Median OS 10 M	□

Notes: OS: overall survival; ELN: European Leukemia Net; NR: not reached; Y: years; M: months.

## Data Availability

None of the patients was involved in the design of this study. The results will be made known to the study participants on the homepage of our website.

## Abbreviations

The following abbreviations are used in this manuscript:

AML	Acute myeloid leukemia
IHC	Immunohistochemical staining
MRP1	Multidrug resistance-associated protein 1
AKR1B10	Aldo-keto reductase family 1 member B10
AKR1B1	Aldo-keto reductase family 1 member B1
AKR1C3	Aldo-keto reductase family 1 member C3
OS	Overall survival
ELN	European Leukemia Net
LBCL	Large B-cell lymphoma
TCL	Aggressive T-cell lymphoma
GRP94	Glucose-regulated protein 94
GRP78	Glucose-regulated protein 78
B-ALL	B-cell acute lymphoblastic leukemia
TGFβ1	Transforming growth factor β1
TNFα1	Tumor necrosis factor α1
TNFR	Tumor necrosis factor receptor
PD-1	Programmed cell death-1
PD-L1	Programmed cell death–ligand 1
ENT1	Equilibrative nucleoside transporter 1
MDR1	Multidrug resistance 1
MRP1	Multidrug resistance-associated protein 1
CYP3A4	Cytochrome P450 3A4
FLT3	fms related receptor tyrosine kinase 3
TKI	Tyrosin kinase inhibitor
CYP2B6	Cytochrome P450 2B6
AKR1C3	Aldo-keto reductase family 1 member C3
AKR1B10	Aldo-keto reductase family 1 member B10
AKR1B1	Aldo-keto reductase family 1 member B1
TP	Thymidine phosphorylase
GST	Glutathione sulfate transferase
APL	Acute promyeloid leukemia
CBF	Core binding factor
CR	Complete remission
PD	Progressive disease
M	Months
NS	Not significant
NR	Not reached
MCL1	Myeloid cell leukemia sequence 1
Y	Years
ER	Endoplasmic reticulum
BCL2	B-cell/CLL lymphoma 2
Del	Deletion
MTX	Methotrexate
C	Cyclophosphamide
OH	Oncovin hydroxyl doxorubicin;
HCCFA	7-hydroxy-2-(4-methoxyphenylimino)-2H-chromene-3-carboxylic acid benzylamide
NSAIDs	N-phenyl-anthranilic acid, diclofenac, and glycyrrhetinic acid
